# Cryptochrome: The magnetosensor with a sinister side?

**DOI:** 10.1371/journal.pbio.3000018

**Published:** 2018-10-02

**Authors:** Lukas Landler, David A. Keays

**Affiliations:** Research Institute of Molecular Pathology, Vienna Biocentre, Vienna, Austria

## Abstract

Over the last three decades, evidence has emerged that low-intensity magnetic fields can influence biological systems. It is now well established that migratory birds have the capacity to detect the Earth's magnetic field; it has been reported that power lines are associated with childhood leukemia and that pulsed magnetic fields increase the production of reactive oxidative species (ROS) in cellular systems. Justifiably, studies in this field have been viewed with skepticism, as the underlying molecular mechanisms are unknown. In the accompanying paper, Sherrard and colleagues report that low-flux pulsed electromagnetic fields (PEMFs) result in aversive behavior in *Drosophila* larvae and ROS production in cell culture. They further report that these responses require the presence of cryptochrome, a putative magnetoreceptor. If correct, it is conceivable that carcinogenesis associated with power lines, PEMF-induced ROS generation, and animal magnetoreception share a common mechanistic basis.

## Abstract 2

This Primer explores recent evidence as to how low-intensity magnetic fields might influence biological systems.

Magnetic fields can influence biological systems, a fact that has been exploited by clinicians to treat disease [1], scientists to study cellular function [2], and by migratory birds to find their way home [3]. Magnetic fields can interact with matter by (1) inducing electric currents, (2) by applying a force on magnetic material, or (3) by influencing chemical reactions [4]. Transcranial magnetic stimulation (TMS), for instance, exploits electromagnetic induction to activate neuronal populations in individuals suffering from Parkinson disease, depression, and motor disorders [5]. In contrast, force-based methods have used magnetic nanoparticles to genetically activate specific neuronal populations, to modulate intracellular trafficking, or to guide cell migration [6–8]. These approaches rely on the application of very strong magnetic fields. In the case of TMS, approved clinical devices apply 1.5T-fields, and force-based magnetogenetic tools rely on the application of 50–500 mT fields [6] (See Fig 1).

**Fig 1:**
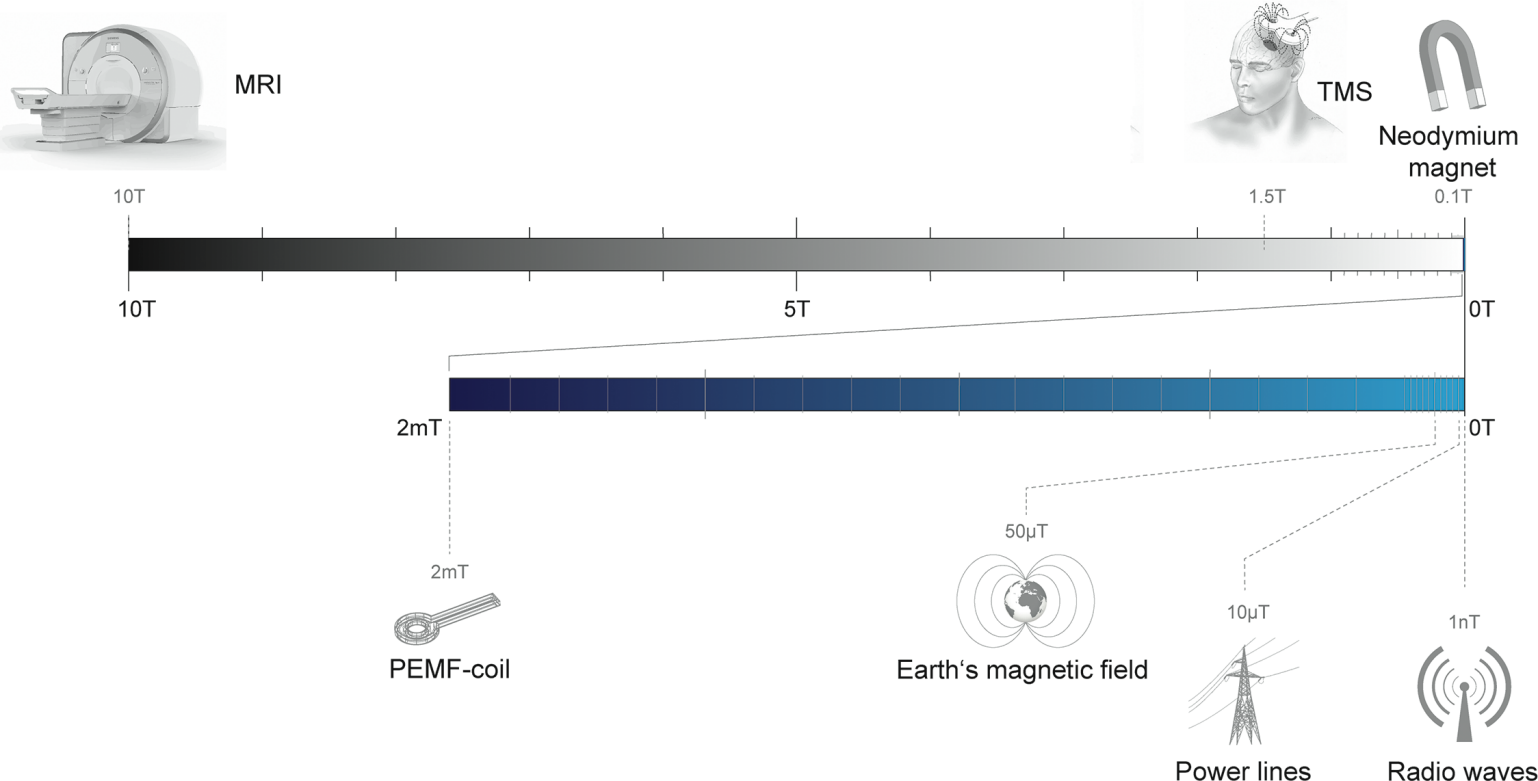
Diagram showing devices that generate magnetic fields and their respective field strengths. To date, most medically relevant magnetic fields are in the Tesla range and used for magnetic resonance imaging (1.5 T–10 T) or transcranial magnetic stimulation (1.5 T). Neodymium magnets produce static fields (100 mT), whereas low-flux PEMFs are in the range of 2 mT and oscillate in the range of 10 to 200 Hz. These fields are considerably stronger than the static field of the Earth (50 μT), oscillating fields generated by powerlines (10 μT, 50 Hz), and radio frequency waves (as low as 1 nT). Please note that the above field strengths should be considered as approximations, as there is considerable variation dependent on the device. PEMF, pulsed electromagnetic field.

What has been unclear for some time is how low-intensity magnetic fields interact with organic molecules. While initially greeted with justified skepticism, there is now considerable evidence showing that this does actually happen. It has been conclusively demonstrated that an array of species on the planet are able to detect earth-strength magnetic fields, a mere 50 μT [9,10]. Within a controlled setting, investigators have been able to manipulate the orientation behavior of European robins [11], loggerhead turtles [12], zebra finches [13], moths [14], mice [15], and ants [16] by changing the magnetic field. Moreover, magnetic orientation in birds, insects, and rodents is perturbed by the application of electromagnetic fields (EMFs) in the nT range, indicative of a highly sensitive sensory apparatus [17–20]. Reflecting the capacity of low-intensity magnetic fields to impact biological systems, a number of studies have implicated EMFs (50 Hz, primarily originating from powerlines) in leukemia, prompting the International Agency for Research on Cancer to classify them as a potential carcinogen [21]. It should be noted, however, that studies on this front have not been unanimous. While Draper and colleagues assessed 29,081 children reporting a significant increase in leukemia in those individuals residing nearby powerlines [22], a study by Sorahan of 73,051 electricity workers in England and Wales reported no association between exposure to magnetic fields and leukemia [23]. These contradictory findings have generated much controversy, which has been amplified by the absence of a clear mechanism that explains how low-flux fields could cause cancer [24].

Similarly, the therapeutic utility and impact of low-flux pulsed electromagnetic fields (PEMFs) has prompted a great deal of discourse. These fields (which are normally in the range of 0–2 mT, oscillating at 10 to 200 Hz) are insufficient to depolarize neurons by electromagnetic induction and have been proposed as treatments for osteoporosis [25], multiple sclerosis [26], Parkinson disease [27–29], and depression [30,31]. In vivo animal studies have claimed that PEMFs increase net calcium (Ca) flux in bones [32], limit osteoarthritis [33], accelerate wound healing [34], stimulate nerve regeneration [35,36], and promote angiogenesis [37]. This has spawned the sale of PEMF devices that can be purchased over the internet that promise to treat a staggering array of unrelated pathologies, energize your red blood cells, and stimulate the body's natural healing process. In making these dubious claims, the retailors of these products rely on a plethora of studies that have analyzed the effects of PEMFs in a cellular context. Through the employment of a variety of different stimulation protocols, cell types, and varying methods of quantitation, it has been reported that PEMFs increase cell proliferation [37,38], induce the expression of bone morphogenetic proteins [39], influence neurite outgrowth [40], reduce neuronal apoptosis [41], and enhance the expression of brain-derived neurotrophic factor [42]. Conversely, studies have also reported that PEMFs cause chromosome aberrations [43], induce the formation of micronuclei [44], and increase the production of reactive oxygen species (ROS) [45]. A major issue associated with this literature is the absence of positive controls, negative controls, and blind quantitation [45]. Appropriate negative controls are the most pressing issue. Some studies employ a sham control in which the magnetic coils are disconnected [46], while others use mu-metal that is placed between the coil and the sample to block the magnetic fields [47]. Such "controls" do not, however, eliminate the potential influence of heat and/or vibrations that are produced by active magnetic coils [48]. With inadequate controls and extravagant claims, it is little wonder that the rational reader is left wondering if any of it is real, and if so, what is the underlying molecular mechanism?

In the accompanying manuscript, Sherrard and colleagues provide some insight on this front with a focus on cryptochromes [49]. These flavoproteins, which are key components of the circadian clock, have been proposed to serve as magnetosensors, as they have the ability to form radical pairs when exposed to light [50]. It is known that radical pairs can exist in either a singlet or triplet state, which can be influenced by the local magnetic environment [51]. According to the prevailing hypothesis, a radical pair is formed in cryptochromes as photoinduced electron transfer occurs along a string of trytophan residues, resulting in the reduction of the flavin adenine dinucleotide (FAD) cofactor. The external magnetic field influences the ratio of radical pairs in the singlet/triplet state, which in turn alters the biochemical properties of the molecule [52]. Consistent with previous studies in *Drosophila*, Sherrard and colleagues demonstrate that an aversive behavioral response in larvae requires the cryptochrome molecule, which can be rescued by ectopic expression of the human homologue [53–56]. Building on this finding, they analyze whether or not PEMFs generate ROS in a *Drosophila* Sf21 cell line, human embyonic kidney (HEK)293 cells, and primary mouse fibroblasts. They report a startling increase in ROS in these cell lines following PEMF exposure that is cryptochrome dependent. A complementary microarray analysis of gene expression in HEK293 cells revealed an enrichment of genes associated with oxidoreductase pathways following PEMF exposure, consistent with the generation of ROS [49].

The critical yardstick in assessing the validity of these claims is an assessment of the controls they employed. This reveals a big improvement on existing papers, but the controls are still imperfect. They employed two different controls for their PEMF stimulation experiments. First, they used a mu-metal sheet (which serves to block the magnetic field), and second, they used a “double-wrapped” coil design. Double-wrapped coils (which should be employed for all experiments that aim to assess the effect of magnetic fields on biological systems) employ two sets of wires wrapped around a single frame [57]. To generate a magnetic stimulus, a current passes through the coil in unison in the same direction, whereas currents running in opposing directions serve as a control. In the latter case, the same heat and vibration is generated, but a magnetic field should be absent. In the case of static fields, the construction of such coils is straight forward, but this is undeniably more challenging in the case of PEMFs. It is important to note that the sham control used by Sherrard and colleagues does not result in a zero magnetic field, but rather, a short (0.01 ms) magnetic pulse of considerable strength (approximately 1.8 mT) remained (see Supplementary Fig 7 in [49]). Nonetheless, this control provides the strongest evidence that the claims made in this paper are valid. Curiously, the short intense magnetic pulse generated by the control coils did not induce ROS production nor did it cause an adverse behavior in *Drosophila* larvae.

Extraordinary claims nonetheless require extraordinary evidence. Should this paper be independently replicated by multiple labs, it will undoubtedly be influential. It is conceivable that leukemia associated with 50 Hz power lines, PEMF-mediated ROS generation, and animal magnetoreception are mechanistically similar—each requiring the presence of cryptochrome. By influencing the spin state of long-lived radicals in the cryptochrome molecule, magnetic fields may influence the generation of ROS, which in turn alters intracellular signaling. With respect to cancerous phenotypes, fields of a particular intensity and frequency may generate higher levels of ROS, causing DNA damage and uncontrolled cell growth (see review of [24]). In support of the aforementioned proposition, there is evidence to suggest that cryptochromes can form ROSs such as hydrogen peroxide (H_2_O_2_) and superoxide (O_2_^−^) following light exposure [58,59], and mammalian Cryptochrome1 may act as a redox sensor within cells, potentially by disulfide bond formation between Cysteine412–Cysteine363 [60]. The extent to which such a mechanism is light dependent is a matter that requires further investigation, particularly given that growing evidence suggests that mammalian cryptochromes do not bind FAD and are not true photoreceptors [24,61].

Finally, the experimental set up described by Sherrard and colleagues may serve as an effective foundation to interrogate the molecular basis of magnetoreception. While there is evidence that cryptochrome is required for magnetic phenotypes, it is still unclear whether it is the actual receptor, what illumination is required, and what signaling pathway it relies on. A cellular system that enables the systematic alteration of lighting conditions, as well as the mutation of different molecules and residues, would be an extremely powerful tool in understanding how magnetic fields influence biological systems. It may well transpire that cryptochrome is a magnetosensor but one with a sinister side.

## Abbreviations

Ca: calcium
EMF: electromagnetic fields
FAD: flavin adenine dinucleotide
H_2_O_2_: hydrogen peroxide
HEK: human embyonic kidney
PEMF: pulsed electromagnetic field
O_2_^−^: superoxide
ROS: reactive oxidative species
TMS: transcranial magnetic stimulation
